# Filling Behavior in Joining Using Pin-like Structures

**DOI:** 10.3390/polym14153083

**Published:** 2022-07-29

**Authors:** Michael Wolf, Dietmar Drummer

**Affiliations:** Institute of Polymer Technology, Friedrich-Alexander-Universität Erlangen-Nürnberg, Am Weichselgarten 10, 91058 Erlangen, Germany; dietmar.drummer@fau.de

**Keywords:** multi-material, adhesion-incompatible, pin-like structures, vibration welding

## Abstract

Multi-material designs enable more efficient use of material-specific properties, which is necessary for sustainable and resource-saving production. However, multi-material polymer joints confront conventional joining methods with major challenges. Therefore, novel joining processes such as joining using pin-like structures are required. Investigations into this innovative process have provided initial findings of, for example, the design criteria of the pin-like structures depending on the material combination. For further optimization of the process, the filling behavior and the shrinkage effects occurring in pin-like joining are herein investigated. These have a decisive influence on the resulting bond quality. To identify the correlations, the joining step was carried out on the one hand using vibration welding technology with and without pre-heating of the structured-partner. On the other hand, the injection molding process was used to realize filling of the structures, as well as cooling under increased pressure. The investigations show that the shrinkage behavior clearly influences the filling degree and the bond properties of the multi-material joint. For shrinkage-intensive materials, filling and cooling under pressure is essential to achieve high mechanical bond strengths, whereas for materials with low shrinkage, the pressure during the joining step is negligible.

## 1. Introduction

Sustainable and resource-saving production is becoming increasingly important [1]. Multi-material designs enable more efficient use of material-specific properties, especially for polymer assemblies, by combining different types of plastics and their specific material characteristics [2]. This not only allows for local part properties (e.g., mechanical, thermal or chemical) but also an extension of the component functionality. For the production of such multi-material combinations, joining processes are required, which have developed into a key technology in the product development cycle [3]. Commonly used joining processes for thermoplastic materials are welding, adhesive bonding and mechanical fasteners [4,5].

However, multi-material joints confront conventional joining methods with major challenges. Most current processes are only partially suitable for joining multi-material systems [6]. In many cases, they are also too restrictive and inflexible to react quickly to the changing requirements when joining dissimilar materials [7]. Mechanical fasteners are limited by the need for free-form surfaces and the joint design (overlap of the joining partners). In addition, there is an increased assembly effort, as well as increased weight due to the fasteners [8], which is counterproductive to resource efficiency and lightweight design. The production of adhesive bonds is time- and cost-intensive due to the surface pretreatment that is usually required [9,10]. In addition, adhesives are often harmful to health [11] and there is no universal adhesive [12]. In most cases, the adhesive must be specifically adapted and developed to the application, the environmental conditions and the material combination. Welding processes, in contrast, are strongly limited in possible material combinations [13]. In addition to similar joining temperatures and similar viscosities at this temperature [14], the necessary adhesion compatibility between the materials significantly limits possible material combinations [13]. Thus, usually only polymers of the same type are joined using welding processes.

**Figure 1:**
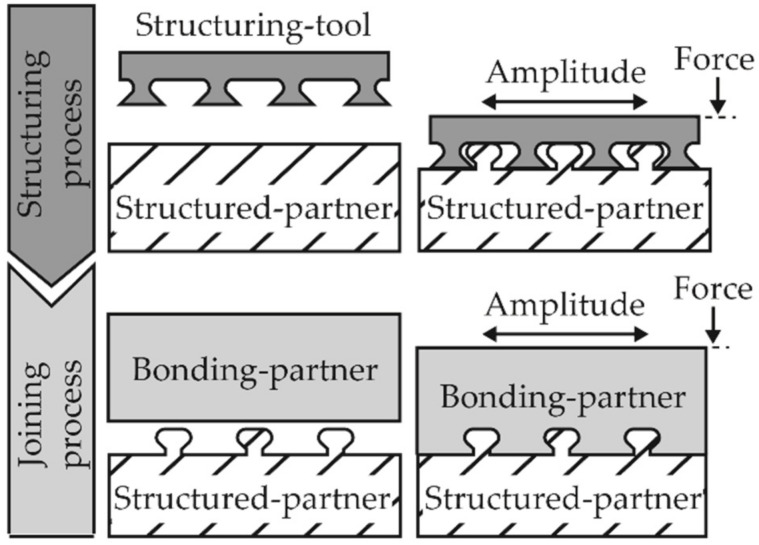
A schematic illustration of the joining using pin-like structures in vibration welding [15].

Due to the existing limitations of conventional joining processes for multi-material joints, novel methods are necessary. One of these novel joining processes is the joining using pin-like structures in the vibration welding process, schematically shown in Figure 1. In the first step, known as the structuring process, pin-like structures are introduced into the structured-partner. A structuring-tool is used, which performs a friction-relative movement with the amplitude *A* and a defined force *F*. The structured-partner melts in the contact zone as a result of the frictional energy introduced, and the pin-like structures are formed. In the second step, the joining process, a friction-relative movement takes place between the bonding-partner and the structured-partner. The bonding partner is plasticized, flows into the cavities between the pin-like structures and thus forms the form-fit connection.

Earlier studies on the joining using pin-like structures were carried out for lap joints between polyamide and polyethylene [15] and butt joints with polypropylene, polymethylmethacrylate and polycarbonate, each in combination with polyamide [16,17,18]. It was shown that high mechanical bond strengths can be achieved between adhesion-incompatible polymer combinations by joining using pin-like structures. The thermo-mechanical material properties of the joining partners must be coordinated in such a way that softening or melting of the pins of the structured-partner during the joining step is avoided [16]. The achievable bond strength, as well as the failure behavior of the joint, depends on the geometry, the dimensions of the pin-like structures and the material combination [17,18]. In addition to a high bond quality, joining using pin-like structures also enables simple and pure separability of the base materials after the product life cycle [19]. Due to the adhesion incompatibility of the materials and thus the absence of material interaction in the joining zone, this can be achieved by simple and material-friendly methods, such as shredding.

Furthermore, it was shown that the filling behavior of the pin-like structures is influenced by the bonding-partner, while the pin structure itself has no, or only a negligible, effect [17]. However, due to the joining step at low pressure, air inclusions at the pin foot area, as well as shrinkage effects, can hinder complete structure filling respectively the resulting filling degree in the vibration welding process. For further improvement, the bond quality and the filling behavior—respectively, the filling degree of the pin-like structures depending on the material and the joining process—is investigated within this study. To achieve an improved filling, as well as to separate the resulting effects and establish general process interactions, the joining step is carried out via different methods; on the one hand, using the vibration welding technique without and with pre-heating of the structured-partner, and on the other hand, by using assembly injection molding with variations of the holding pressure.

## 2. Materials and Methods

### 2.1. Materials and Test Specimen

Polyamide 66 (PA66), type Ultramid A3K (BASF AG, Ludwigshafen, Germany) was used as the structured-partner. The semi-crystalline material polypropylene (PP), type Sabic 505P (Sabic Europe B.V., Sittard, The Netherlands), as well as the amorphous thermoplastic polymethylmethacrylate (PMMA), type Plexiglas 7N (Evonik Industries AG, Essen, Germany) were used as bonding-partner.

For the investigations, plates with the dimensions of 115 × 115 × 4 mm^3^ were produced by injection molding with a machine of the type Allrounder 370V/800-315 (Arburg GmbH & Co.KG, Lossburg, Germany). Before the processing, the PA66 granulate was dried at 80 °C for 4 h. The injection molding processing parameters were defined according to the manufacturer specifications. Subsequently, test specimens with the dimensions of 52 × 50 × 4 mm^3^ were prepared by milling.

### 2.2. Material Analysis

The rheological behavior of the bonding-partners was investigated during cooling by rotational viscometry. The aim was to determine the temperature at which the material is no longer flowable. Therefore, the cross-over point between the loss and storage modulus can be evaluated. This intersection represents a limit below which elastic material behavior dominates [20]. Thus, flow processes are only possible to a limited extent from here on. Due to the very low shear stress during filling of the pin-like structures, the measurements were carried out at a frequency of 1 Hz at a cooling rate of 3 K/min. A rheometer type AR 2000 (TA Instruments, New Castle, DE, USA) was used in oscillating plate-to-plate measurements on specimens with a diameter of 25 mm.

The shrinkage behavior of the bonding-partner (PP and PMMA) was analyzed using isobaric pvT measurements. Therefore, a pvT-device of the type Rheograph 25 (Göttfert Werkstoff Prüfmaschinen GmbH, Buchen, Germany) was used. The shrinkage behavior was evaluated at 20 bar, 300 bar and 500 bar. 20 bar is approximately the pressure range that is present during the joining by means of the vibration welding technology. 300 bar and 500 bar are used as holding pressure in injection molding. With the help of this analysis, correlations between the shrinkage and the resulting structural filling shall be enabled. The specific volume at the defined pressures was calculated using the modified two-domain Tait Equation (1). Where *V(T,P)* is the specific volume, *V*_0_ is the specific volume at zero pressure, *T* is the temperature, *P* is the pressure, *C* is a constant (0.0894) and *B* accounts for the pressure sensitivity of the material. For non-amorphous materials, an additional transition function is required, described by Equation (2).

(1) $$V\left(T,P\right)=V_{0}\left(T\right)\times \left[1-C\;ln\left(1+\frac{P}{B\left(T\right)}\right)\right]+V_{t}\left(T,P\right)$$

(2) $$V_{t}\left(T,P\right)=b_{7}exp\left[\left(b_{8}\left(T-b_{5}\right)\right)-\left(b_{9}\;P\right)\right]$$

The upper temperature region (*T > Tt*) can be described by:

(3) $$V_{0}=b_{1m}+b_{2m}\left(T-b_{5}\right)$$

(4) $$B\left(T\right)=b_{3m}exp\left[-b_{4m}\left(T-b_{5}\right)\right]$$

The lower temperature region (*T < Tt*) can be described by:

(5) $$V_{0}=b_{1s}+b_{2s}\left(T-b_{5}\right)$$

(6) $$B\left(T\right)=b_{3s}exp\left[-b_{4s}\left(T-b_{5}\right)\right]$$

### 2.3. Joining Trials

**Figure 2:**
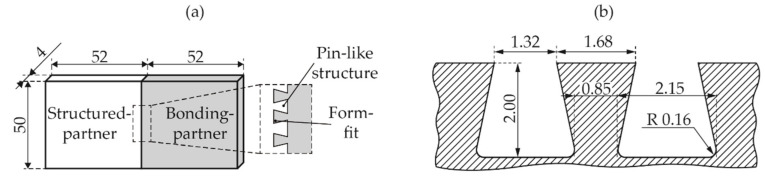
A schematical illustration of (**a**) adhesion-incompatible multi-material butt joint geometry, and (**b**) main geometrical dimensions of structuring-tool geometry.

The test specimens were used to produce the adhesion incompatible butt joints based on a form-fit, shown in Figure 2a. The PA66 (structured-partner) was structured for all variants using a laboratory welding machine of the type Branson M-112 HR (Branson Ultraschall, EMERSON Technologies GmbH & Co. KG, Dietzenbach, Germany). The structuring-tool with dimensions of 50 × 50 × 4 mm^3^ has 16 trapezoidal pin structures with a height of 2.0 mm in the contact area with the structured-partner. The main geometrical dimensions can be seen in Figure 2b, and a detailed drawing of the trapezoidal tool can be seen in [17]. The structuring parameters were chosen analogues with previous investigations [17,18,19], with a frequency of 235 Hz, an amplitude of 0.7 mm, a structuring force of 600 N and a structuring path of 1.2 mm. Before both process steps, structuring and joining, the PA66 specimens were dried in a vacuum oven at 70 °C until weight constancy was achieved according to DIN EN 1110 [21].

The second process step, the joining, was carried out on the one hand using the vibration welding process with the same machine as in the structuring step. The process parameters were selected analog to previous investigations [17,18,19]: frequency 235 Hz, amplitude 0.7 mm, joining force 400 N, joining path 1.5 mm. The structured-partner had ambient temperature (approximately 25 °C) in the joining step. In addition, tests were carried out with pre-heated structured-partners. For this purpose, the structured-partner was heated in a heating oven and held isothermally until a homogeneous heat penetration was achieved. The joining step was started subsequently at a defined temperature of 80 °C and 120 °C. These temperatures were selected based on the thermal material properties of the used joining partners. Both temperatures are well below the softening area (above 200 °C) of the structured-partner of PA66. The pre-heating temperature of 80 °C is below the glass transition temperature of PMMA (approximately 100–110 °C) and slightly below the beginning of the softening area of PP (above approximately 90 °C). The second pre-heating temperature of 120 °C is just above these two levels. The temperature was monitored using thermocouples type K. The pre-heating of the structured-partner should result in slower cooling of the molten bonding-partner, and in particular, in a longer flow of the material and thus better filling of the pin-like structures.

On the other hand, butt joints were produced by injection molding with a machine of the type Allrounder 370V/800-315 (Arburg GmbH & Co.KG, Lossburg, Germany). The structured-partner was inserted into the mold cavity and the bonding-partner was injected. The process parameters were selected according to the manufacturer’s specifications, and the main parameters are shown in Table 1. The tests were carried out with two different holding pressures. Compared with the conventional joining step using the vibration welding process, the clearly higher injection pressure results in an optimal filling of the pin-like structure. In contrast to the conventional joining step, the holding pressure counteracts shrinkage effects.

### 2.4. Optical Characterization

The resulting filling of the pin-like structures was analyzed by means of computer tomography (CT) investigations. A CT device of the type subμ-CT (Fraunhofer-Institute for Integrated Circuits (IIS) e. V., Erlangen, Germany) was used. To ensure a high resolution, specimens with dimensions of 12 × 20 × 4 mm^3^ were prepared for the optical analysis. The degree of filling was analyzed three-dimensionally by means of the evaluation software Modular Algorithms for Volume Images, MAVI version 1.5.2 (Fraunhofer Institute for Industrial Mathematics (ITWM) e. V., Kaiserslautern, Germany). Due to the minor density difference of PA66 and PMMA, the software-based evaluation could only be carried out for the combination PA66-PP. Consequently, the filling behavior for all joining variants was additionally analyzed using two-dimensional CT images. Due to the theoretically two-dimensional form of the pin-like structures and the joint exclusively based on form-fit in the area of these structures, 2D CT images should be able to describe the multi-material joint sufficiently. For this purpose, the cavity area between the pin-like structures was measured and taken as the total volume, which is to be filled. Subsequently, the unfilled horizontal area in the bottom cavity area, as well as the unfilled vertical area at the two pin flanks, were evaluated. Due to the almost equal flow conditions at each pin flank, an identical filling was assumed for the two pin flanks that are not visible in the 2D image, as well as for the two visible flanks. The total sum of the unfilled areas could be estimated under this assumption and related to the total cavity volume. The consistency of this two-dimensional analysis of the CT images with the three-dimensional evaluation of the volume fractions is validated on the PA66-PP connection.

### 2.5. Mechanical Behavior

Tensile tests with a pull-off speed of 1 mm/min were carried out under standard conditions according to DIN EN ISO 291 [22] (T = 23 °C, relative humidity 50%) to evaluate the bond quality. A universal testing machine via computer-controlled test execution of the type Zwick 1448 (Zwick GmbH & Co.KG, Ulm, Germany) was used. The number of repetitions was five. Before testing, the multi-material joints were stored in a vacuum oven at 23 °C to prevent moisture absorption after joining.

## 3. Results and Discussion

### 3.1. Rheological Behavior of Bonding-Partner

**Figure 3:**
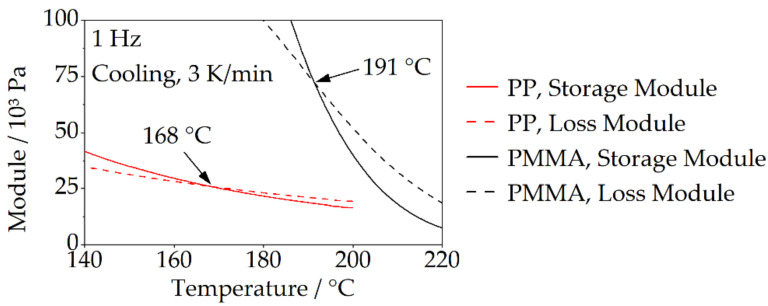
The cross-over point during the cooling of bonding-partners.

Figure 3 shows the loss and storage modulus curves of the two bonding-partners during cooling determined by rheological tests. The intersection of the two curves—the cross-over point—represents a characteristic limit below which an elastic material behavior dominates [20], and consequently, flow processes can only occur to a limited extent. It can therefore be assumed that shrinkage effects occurring below the cross-over point can no longer be compensated by flow processes. The cross-over point for PP is at 168 °C, and thus well above the crystallization temperature determined by DSC in the range of 120 °C. For PMMA, an elastic material behavior with clearly reduced flowability dominates below a temperature of 191 °C. In the following, the temperature of the cross-over point is used for the evaluation of the shrinkage behavior.

### 3.2. Shrinkage Characteristics

**Figure 4:**
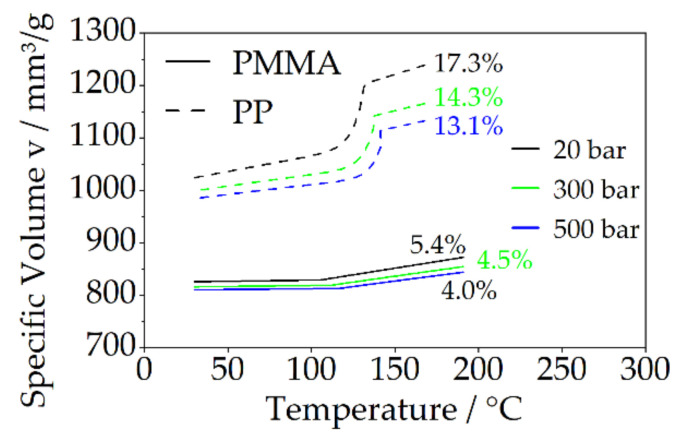
The shrinkage behavior of the materials used as bonding-partner using TAIT model.

The shrinkage behavior of the bonding-partners was evaluated at a pressure of 20 bar, 300 bar and 500 bar in the temperature range between the cross-over point (Figure 3) to ambient temperature, shown in Figure 4. Between the two bonding-partners, a clearly different shrinkage behavior can be observed. While PMMA exhibits only a slight decrease in specific volume at 5% due to its amorphous structure at 20 bar, the volume reduction for the semi-crystalline PP is clearly higher at about 17%. At higher pressures of 300 bar and 500 bar (used holding pressure level), the shrinkage of PP decreased to 14% (300 bar) and 13% (500 bar), respectively. Nevertheless, the shrinkage is still clearly higher than for PMMA, which shows only a marginal influence of the volume reduction by the increased pressure in the investigated area.

Due to the low pressure during the filling of the pin-like structures, as well as the cooling phase, shrinkage effects cannot be compensated to a sufficient extent in the pin-like joining using the vibratory welding process. The clearly greater decrease in specific volume for PP (almost 3.5 times higher as PMMA) leads to the expectation of greater shrinkage effects and thus possibly larger hollows or gaps in the pin area for the material combination PA66-PP, compared to PA66-PMMA. At pressures of 300 bar and 500 bar, shrinkage effects still occur during the cooling phase of the injection molding process. However, it can be expected that these effects will be compensated to a certain degree by the holding pressure, and thus an increased structural filling will be achieved.

### 3.3. Filling Behavior

**Figure 5:**
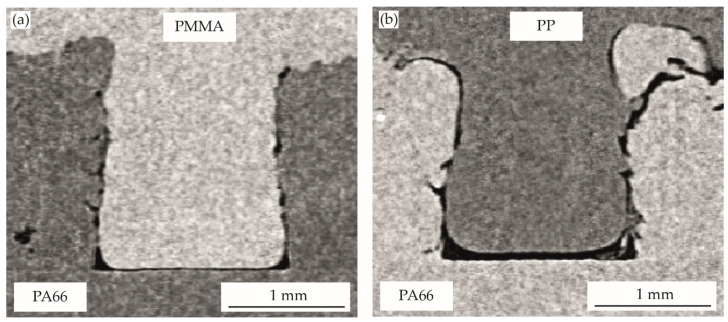
The filling of pin-like structures in vibration welding technology without pre-heating:(**a**) PA66-PMMA, (**b**) PA66-PP.

**Figure 6:**
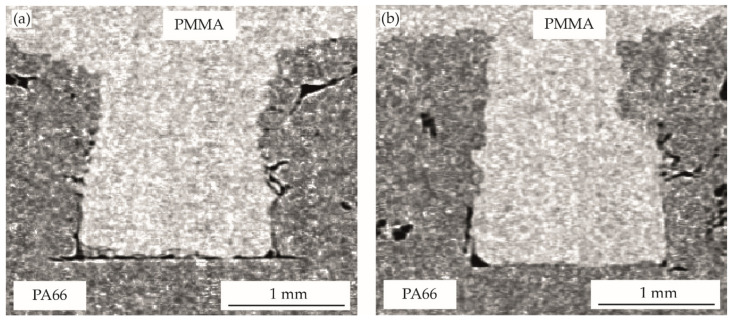
The filling of pin-like structures in vibration welding technology for PA66-PMMA joints and pre-heating of PA66 to: (**a**) 80 °C, (**b**) 120 °C.

**Figure 7:**
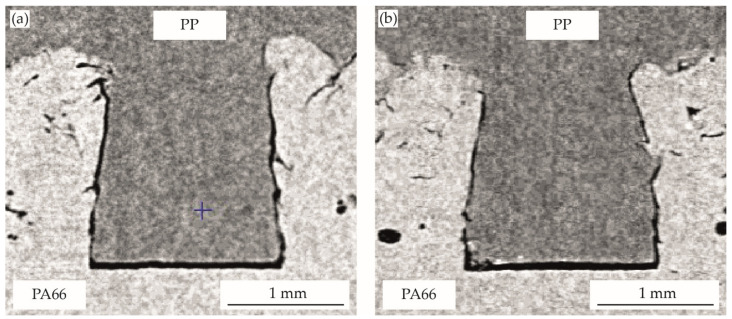
The filling of pin-like structures in vibration welding technology for PA66-PP joints and pre-heating of PA66 to: (**a**) 80 °C, (**b**) 120 °C.

**Figure 8:**
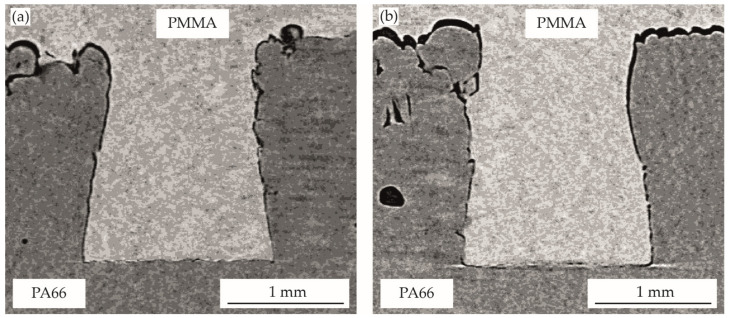
The filling of pin-like structures in injection molding for PA66-PMMA joints with holding-pressure of: (**a**) 300 bar, (**b**) 500 bar.

**Figure 9:**
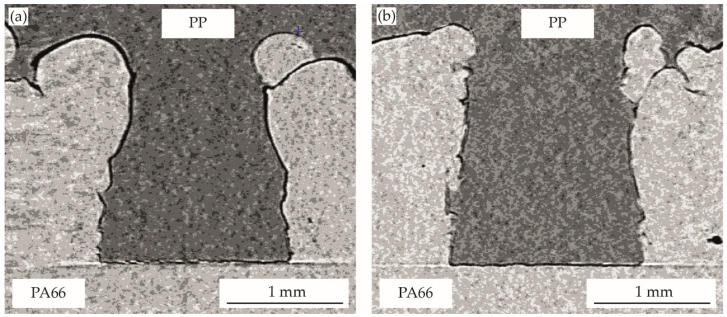
The filling of pin-like structures in injection molding for PA66-PP joints with holding-pressure of: (**a**) 300 bar, (**b**) 500 bar.

The optical evaluation of the filling behavior of the pin-like structures is shown in Figure 5, Figure 6, Figure 7, Figure 8 and Figure 9. Table 2 gives the analyzed unfilled areas in percentage of the form-fit connection for all process variants. There are only minor differences between the evaluation methods. On the one hand, these differences are due to the approximation of the unfilled areas at the invisible pin flanks and the analysis of only one sectional plane for the two-dimensional CT images. On the other hand, gray level variations in the transition areas of both materials may also slightly influence the three-dimensional MAVI evaluation. Due to the very similar results and identical trends, further discussion will be based on the two-dimensional analysis of the CT images, as results were generated for all variants here.

In the conventional joining step using vibration welding technology without pre-heating of the structured-partner (Figure 5), an almost complete structure filling is shown for the bonding-partner PMMA. Unfilled areas are only visible in the pin-foot area (gap of approximately 10–20 µm and air inclusions in the edge areas) and slightly on the lateral pin surface. The evaluation of the structure filling (Table 2) shows a filling of approximately 93% of the generated pin cavity (unfilled area: 7.2%). In comparison, the multi-material bond with PP as the bonding partner clearly shows that more unfilled areas (13.1%), as well as a structure filling of approximately 87%, are achieved. The unfilled areas are located on the lateral pin surfaces and in particular in the lower pin foot area. Here, a gap of about 60 µm is visible.

The results show that the filling behavior is clearly dependent on the bonding-partner. For both variants, PP and PMMA, a non-complete filling can be assumed during the joining step, as can be seen by the rounded edge areas of the flow front. The degree of filling achieved is significantly higher for PMMA. This is attributable to lower shrinkage effects as well as to the temperature range in which flow can take place. This temperature range is very small for PP, between approximately 174 °C (joining temperature [16]) and about 168 °C (Figure 3), whereas for PMMA, flow can occur between approximately 228 °C (joining temperature [16]) and 190 °C (Figure 3). A difference in the structure filling caused by the flowability of the bonding-partner is not to be expected for the two selected materials. PP and PMMA have a similar viscosity level in the flow range, whereby the complex viscosity for PP is even at a slightly lower level (PP: approximately 400–500 Pa s, PMMA: approximately 600–1000 Pa s [16]).

The achievable cavity filling when the structured-partner is pre-heated to 80 °C or 120 °C during the joining step is shown for PMMA in Figure 6 and PP in Figure 7. For the bonding-partner PMMA, an improved filling of the structure is visible, especially for the higher pre-heating temperature. The air inclusions in the edge areas of the pin foot could be reduced and a filling of 97.4% was achieved at a pre-heating temperature of 120 °C (93.6% for pre-heating temperature of 80 °C). In contrast, a surrounding gap can be seen for the bonding-partner PP even when the structured-partner is pre-heated. With a thickness of 50–55 µm, the gap in the pin foot area is smaller than for the multi-material bond without pre-heating. The pre-heating slightly increases the degree of filling to 87.8% (80 °C pre-heating) and 90.9% (120 °C pre-heating). Striking is the shape of the cavity filling in the lower pin area. While the PP flow front is rounded without pre-heating of the structured-partner, these corner areas have sharp edges when the PA66 is pre-heated, independent of the pre-heating temperature. Further, the PP flow front reproduces the surface structure of the PA66 more accurately with pre-heating. The changed flow front in combination with the clear reproduction of the PA66 surface structure indicates a complete structure filling during the joining step with subsequent shrinkage. The improved filling can be explained by a reduced cooling rate during the filling of the cavity. Cooling of the melt formed in the joining zone begins directly as it flows into the cavity area. Due to the lower temperature difference caused by the pre-heated structured-partner, the melt cools slower and thus remains flowable for a longer time. During solidification and cooling, shrinkage effects result that cannot be compensated for due to the low pressure level in the vibration welding process and a missing melt layer. As a result, the volume of the PP decreases, resulting in a clearly visible gap between the two materials.

Compared to the pvT model, the unfilled volume of the multi-material joint is in the range of the evaluated volume shrinkage of the bonding-partner. The clearly increased shrinkage of the semi-crystalline PP (compared to PMMA) in the pvT is also evident for the form-fit joint. The slightly lower values for the multi-material connection with pre-heated structured-partner (here complete filling of the cavity during joining can be assumed) compared to the pvT can be explained by the simplifications made. The pressure present in the cavity area between the pin-like structures can be higher than the assumed pressure of 20 bar. This pressure is effective in the joining zone, referred to the total contact area of the two partners. Due to the reduced cavity area, where melt flow takes place, a higher pressure can exist locally. Furthermore, the cross-over point was determined in a rotational viscometer at low shear. The effective shear stress in the pin area may be increased, which would lead to a shift of the flow area to lower temperatures. Both would result in a reduction in shrinkage. Finally, during cooling of the pre-heated structured-partner, a change in length takes place due to the existing thermal expansion. This slightly reduces the volume of the cavity area compared to the filling process, which in turn results also in reduced shrinkage effects. Nevertheless, the trends evaluated are in agreement, regardless of the simplifications made.

The optical characterization of the multi-material bond produced by assembly injection molding is shown for the bonding-partner PMMA in Figure 8 and for PP in Figure 9. For both bonding-partners, the result is an almost complete filling of the cavity. The holding pressure level shows no significant influence in the investigated area. For all variants, a filling degree of approximately 93–96% (Table 2) is achieved and the negative structure of PA66 is exactly reproduced by the bonding-partner. Despite the clearly different volume shrinkage of the two bonding-partners (Figure 4), the filling levels is in a similar range. Shrinkage effects can be compensated in a wide range by the higher pressure during cooling in the injection molding process, as comparisons with the pvT measurement at 300 bar and 500 bar show, especially for the semi-crystalline PP.

For all variants, a surrounding, small gap can be seen between the two materials.

In contrast to the joints produced in the vibration welding process, a gap is also visible in the area of the PA66 pin head in injection molding. Due to the clear reproduction accuracy of the surface structure of the PA66 in all areas, complete filling in the joining step and subsequent shrinkage during cooling can be assumed. In areas with a sharp undercut, the gap is smaller than in areas without vertical undercut. The undercut structures prevent shrinkage of the bonding partner due to mechanical interlocks, whereas on free surfaces a clearly lower resistance to volume shrinkage is present. At the PA66 pin head area, unhindered shrinkage takes place. Thermal expansion effects during cooling of the PA66 component from mold to ambient temperature can also have a slight influence on the resulting gaps. The clear difference in the PA66 pin head area between the two joining methods (injection molding or vibration welding) can be attributed to the aggregate condition of the bonding-partner during the joining step. While in the vibration welding process the bonding-partner is in solid form and is only plasticized in the contact zone with the structured-partner, in the injection molding process the bonding-partner is completely molten and solidifies after the pin-like structure has been filled. Due to this increased melt volume in the pin head area, shrinkage effects are much more pronounced at this area. However, this presumably affects the mechanical properties of the multi-material connection only negligibly, since there are no undercuts in the area with increased shrinkage and thus the form-fit connection is not limited.

In addition to the rheological material properties, investigations show that shrinkage effects mainly influence the filling degree. Especially for bonding-partners with high shrinkage, these cannot be compensated for in the conventional joining step using vibration welding technology (with as well as without pre-heating) due to the low pressure level. Here, comparatively wide, unfilled areas result, which presumably will affect the bond properties as well.

### 3.4. Bond Properties

**Figure 10:**
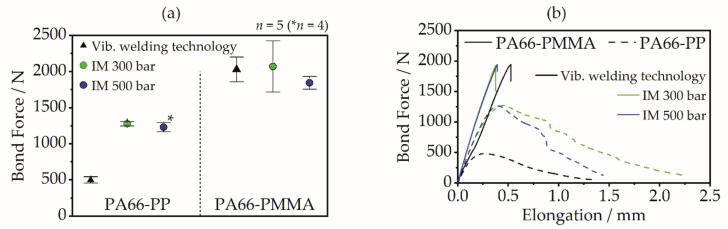
The bond properties of the multi-material connection using pin-like structures: (**a**) evaluated bond force, (**b**) exemplary force-elongation curves.

The mechanical bond properties of the multi-material joints produced by vibration welding technology without pre-heating and by assembly injection molding are shown in Figure 10. Regardless of the manufacturing process, the PA66-PMMA joint exhibits a bond force in the range of 2000 N. For the PA66-PP combination, a clear difference can be seen depending on the joining step. While joints produced by assembly injection molding, irrespective of the holding pressure level, exhibit bond forces in the range of 1200–1300 N, the bond force for joints produced by vibration welding is significantly lower, at approximately 500 N. This can be attributed to the degree of filling of the structures achieved in the joining step. While for PMMA as a bonding-partner, similar filling degrees and, in particular, also a filling of the pin-foot area, can be achieved for both joining variants, for PP a clearly reduced filling takes place using vibration welding technology. Especially along the pin sides and in the bottom edge areas of the pins, unfilled areas remain for the PA66-PP combination. These reduce the resulting undercut area, which means that the theoretical form fit can only be partially utilized. The fracture behavior of the multi-material joint based on pin-like structures also clearly shows the influence of filling behavior. For joints between PA66 and PMMA (high degree of filling for all variants), cohesive failure by fracture takes place throughout. The fracture occurs for injection-molded joints always and for vibration-welded joints predominantly at the pin-foot area of the PMMA components. In contrast, the PA66-PP joint shows adhesive failure by pull-out of the PP from the pin gaps of the PA66 when vibration welding technique was used. For the increased degrees of filling (similar to those of the PA66-PMMA joints) achieved by injection molding, a predominantly cohesive failure occurs due to fracture of the PP component in the pin-foot area. The material strength can thus be fully utilized for the underlying pin geometry due to the increased filling behavior.

The mechanical characterization thus agrees well with the optical analysis of the filling behavior. In general, it can be stated that for high-shrinkage bonding-partners (usually semi-crystalline thermoplastics), filling and cooling under increased pressure has advantages with regard to the degree of filling and, as a result, to the bond quality. For bonding-partners with low shrinkage (e.g., amorphous thermoplastics), the pressure during the filling process is negligible for the bond quality as soon as a complete filling of the structure is achieved.

## 4. Conclusions

The investigations show that, in addition to the geometric design of the pin-like structures and the interaction of the thermo-mechanical material properties of the joining partners, the filling behavior and, in particular, shrinkage effects influence the achievable bond quality. Due to the joining and cooling under low pressure when using vibration welding technology, shrinkage effects of the bonding-partner cannot be compensated. In the case of high-shrinkage materials, typically semi-crystalline thermoplastics, this leads to a gap formation and thus to a reduced utilization of the undercut area of the pin-like structures. The mechanical bond properties are thus reduced. Pre-heating of the structured-partner improve the filling of the structures, as can be seen from the clear reproduction of the negative structure of the structured-partner and the sharp flow front. However, shrinkage effects can only be reduced slightly for semi-crystalline thermoplastics. By applying a higher pressure level in the joining step, reproduced in the injection molding process, it was possible to achieve almost complete structure filling even for the high-shrinkage bonding-partner PP, and to clearly increase the bond strength of this multi-material connection. For low-shrinkage bonding-partners, such as amorphous thermoplastics, the pressure during the joining step is negligible. Due to only a very slight decrease in volume during cooling, high structural filling and high bond properties can be achieved even at low pressure.

As a general conclusion, it can be stated that shrinkage effects in joining by means of pin-like structures have a negative effect on the bond quality and have to be avoided. This can be achieved through different methods: Materials with low shrinkage can be used as bonding-partners. Otherwise, the joining step and cooling can be carried out under increased pressure, or the shrinkage of the bonding-partner can be reduced by using fillers (such as talcum). When using higher pressure levels, however, it must be ensured that this pressure also reaches the foot-area of the structure in order to be able to adequately counteract the volume reduction.

## Figures and Tables

**Table 1:** The main injection molding parameters for a multi-material joint (selected according manufacture’s specifications).

Bonding Partner	PMMA	PP
Mass temperature [°C]	230	250
Mold temperature [°C]	60	60
Injection speed [mm/s]	60	60
Holding pressure [bar]	300/500	300/500
Holding time [s]	20	20

**Table 2:** An evaluated filling degree of the pin-like structures (*n* = 3).

Bonding-Partner	Filling Process		Unfilled Area [%]	
			3D MAVI Evaluation	2D CT Image Measurement
PP	Joining with vibration welding technology	Conventional	14.5 ± 0.9	13.1 ± 0.7
		Pre-heating 80 °C	11.8 ± 0.5	12.2 ± 0.7
		Pre-heating 120 °C	8.2 ± 0.8	9.1 ± 0.8
	Injection molding with holding-pressure of:	300 bar	6.0 ± 0.3	6.7 ± 0.4
		500 bar	5.6 ± 0.1	3.9 ± 0.2
PMMA	Joining with vibration welding technology	Conventional	-	7.2 ± 0.3
		Pre-heating 80 °C	-	6.6 ± 0.7
		Pre-heating 120 °C	-	2.6 ± 0.2
	Injection molding with holding-pressure of:	300 bar	-	5.3 ± 0.1
		500 bar	-	4.5 ± 0.5

## Data Availability

Not applicable.
